# Toward a Global Phylogeny of the “Living Fossil" Crustacean Order of the Notostraca

**DOI:** 10.1371/journal.pone.0034998

**Published:** 2012-04-18

**Authors:** Bram Vanschoenwinkel, Tom Pinceel, Maarten P. M. Vanhove, Carla Denis, Merlijn Jocque, Brian V. Timms, Luc Brendonck

**Affiliations:** 1 Laboratory of Aquatic Ecology and Evolutionary Biology, KU Leuven, Leuven, Belgium; 2 Laboratory of Animal Diversity and Systematics, KU Leuven, Leuven, Belgium; 3 Royal Belgian Institute of Natural Sciences, Brussels, Belgium; 4 Australian Museum, Sydney, New South Wales, Australia; Brigham Young University, United States of America

## Abstract

Tadpole shrimp (Crustacea, Notostraca) are iconic inhabitants of temporary aquatic habitats worldwide. Often cited as prime examples of evolutionary stasis, surviving representatives closely resemble fossils older than 200 mya, suggestive of an ancient origin. Despite significant interest in the group as ‘living fossils’ the taxonomy of surviving taxa is still under debate and both the phylogenetic relationships among different lineages and the timing of diversification remain unclear. We constructed a molecular phylogeny of the Notostraca using model based phylogenetic methods. Our analyses supported the monophyly of the two genera *Triops* and *Lepidurus*, although for *Triops* support was weak. Results also revealed high levels of cryptic diversity as well as a peculiar biogeographic link between Australia and North America presumably mediated by historic long distance dispersal. We concluded that, although some present day tadpole shrimp species closely resemble fossil specimens as old as 250 mya, no molecular support was found for an ancient (pre) Mesozoic radiation. Instead, living tadpole shrimp are most likely the result of a relatively recent radiation in the Cenozoic era and close resemblances between recent and fossil taxa are probably the result of the highly conserved general morphology in this group and of homoplasy.

## Introduction

Tadpole shrimp (Crustacea, Notostraca) comprise one living family, the Triopsidae, including two genera: *Triops* Schrank, 1803 and *Lepidurus* Leach, 1819. Members of this group are often considered prime examples of evolutionary stasis [1]–[3] with the oldest confirmed notostracan fossils dating back as far as the Upper Carboniferous period [4]. Alleged to have remained virtually unchanged during an evolutionary timeframe of more than 250 million years, some surviving members of this ancient crustacean order are frequently referred to as living fossils. The contemporary *Triops cancriformis* (Bosc, 1801), for instance, is regularly cited as the oldest living species because of its striking resemblance to late Permian [5] and early Triassic fossils [6]–[10]. Similarly, fossils from the late Cretaceous have been identified as the living species *Triops longicaudatus* (le Conte, 1846) while other fossils from similar deposits of the same age were classified in the extant genus *Lepidurus* [11], [12]. The long evolutionary history of the group, together with its presumed ‘living fossil’ status and wide current distribution ranges, are suggestive of an ancient radiation.

Tadpole shrimp have a near-worldwide distribution with highest abundances in arid and semi-arid regions [13]. Both genera are typical for freshwater, and occasionally saline, temporary aquatic habitats although certain *Lepidurus* species have been recorded in permanent lakes in arctic regions [14]. They are opportunistic predators, surviving unfavorable conditions such as drought or frost as dormant eggs in the sediment [15]. While both genera can be morphologically distinguished by the presence of a supra-anal plate in *Lepidurus*, tadpole shrimp are known to display substantial levels of within-species phenotypic plasticity. In contrast with a highly conserved general morphology, notostracans typically show considerable phenotypic variation within lineages and populations making it difficult to distinguish species and subspecies [13], [16]. Within *Triops*, for instance, the absence of second maxillae is a good diagnostic character to distinguish *T. australiensis* (Spencer & Hall, 1895) and *T. longicaudatus* from *T. cancriformis* and *T. granarius* (Lucas, 1864). However, variation in other morphological traits such as telson armature, number of segments and shape of the dorsal organ is often less consistent. Consequently, various authors suggest that morphological taxonomy should be handled with utmost care, considering large numbers of individuals [13], [17], [18]. Further complicating systematics are the different modes of reproduction that evolved within the notostracans. Depending on species and population, gonochoric (separate sexes), hermaphroditic as well as androdioecious populations (containing hermaphrodites and a proportion of males) are found [19], [20].

In the 1950's, Linder [17] and Longhurst [21] revised the alpha taxonomy of the Notostraca reducing the number of accepted nominal species from more than fifty to four in *Triops* and five in *Lepidurus*. Based on molecular phylogenies, however, it was recently proposed to recognise more species, even though molecular divergence among clades is often quite low [22]. At this time there are six accepted *Triops* species with presumably four additional lineages deserving species status [22] and approximately 8 *Lepidurus* species [3], [16].

Currently, molecular phylogenetic research is almost exclusively limited to representatives of *Triops*, but see [3], [23]–[24], and large-scale studies considering large numbers of populations over a significant proportion of species distributions are rare [22], [25]. Except for a first exploratory study by Mantovani and coworkers [26], no attempt has been made to reconstruct the phylogenetic relationships within this group at a global scale and considering most recognised species and subspecies. The main reason is that in contrast to the well studied *T. cancriformis* and *T. mauritanicus* (Ghigi, 1921) populations in Europe, material from less intensively studied continents such as Africa, South America and Australia was not available.

Here, we use DNA sequence data from two mitochondrial genes (the protein coding Cytochrome c Oxidase subunit I or COI and 12S rRNA) to elucidate the evolutionary relationships between notostracans from 60 different populations around the globe. Available sequence information is combined with a large number of newly obtained sequences, featuring several recently discovered Australian notostracan lineages. Due to the large scale of the study and the isolated nature of the considered populations, it is reasonable to assume that gene flow will be extremely low and matrilineal markers will suffice to gain insight in phylogenetic relationships at this scale.

Based on this dataset we evaluate the monophyly of both recognised genera, discuss the biogeography and phylogenetic relations among extant lineages and evaluate the potential presence of cryptic species in the light of the often controversial species delineations in notostracans. Since discussion of species status requires a taxonomic revision including morphological studies, rather than going into the taxonomic status of closely related species complexes, we focus on major evolutionary lineages. Finally, we use molecular clocks to investigate whether gene genealogies are consistent with an ancient (pre) Mesozoic radiation suggested by fossil remains.

## Results

**Figure 1:**
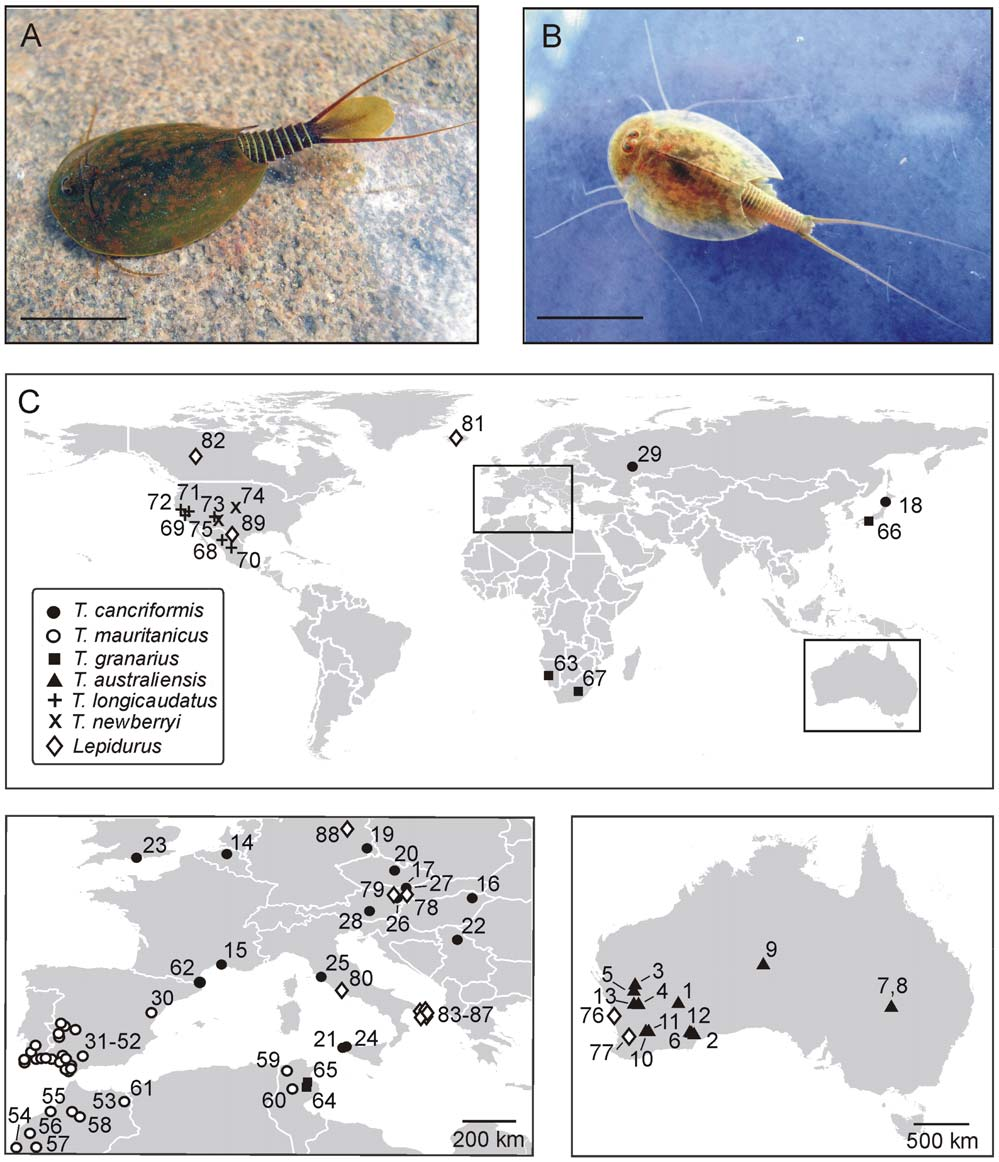
Overview of the general habitus and the geographic distribution of Notostraca taxa and populations included in this study. (A–B) Examples of tadpole shrimp representatives belonging to the genera *Lepidurus* and *Triops*, respectively, illustrating the supra anal plate: a posterior extension of the telson characteristic for *Lepidurus*. (A) *Lepidurus apus* (photo: Jacques Pages), (B) *Triops cancriformis* (photo: Aline Waterkeyn), scale bar = 2 cm; (C) Geographic distribution of investigated Notostraca populations. Locality numbers correspond with population entries in Table S1.

An overview of the 89 *Triops* and *Lepidurus* populations included in our analyses and their localities is provided in Table S1 and plotted in Figure 1. Detailed information about the known distribution of different Notostracan lineages can be consulted in Text S1.

### Characteristics of the mitochondrial DNA sequences and alignment

41 COI and 53 12S sequences were aligned together with 123 additional COI and 74 12S sequences drawn from GenBank and trimmed to a length of 568 bp and 328 bp, respectively. Excluding the outgroup, the complete COI and 12S datasets comprised 78 and 72 unique haplotypes, respectively. The COI alignment contained 240 variable sites (42%) of which 223 (39%) were found to be parsimony informative while the 12S alignment contained 111 variable sites (34%) of which 97 (29%) were parsimony informative.

### Genetic distances and mitochondrial DNA diversity

A COI maximum K2P distance of 35.7% was recorded between an Australian *T. australiensis* haplotype and *L. couesii* from Canada while the maximum 12S distance of 27.8% was calculated between South African *T. granarius* and *T. cancriformis* from Belgium. An overview of the average, minimum and maximum K2P distances within and among main notostracan lineages is provided in Table 1 and Table S2. Estimates of divergence times between main lineages are provided in Table S3. An additional genetic distance matrix calculated using uncorrected p distances is provided in Table S4.

**Table 1:** Molecular divergence (minimum, maximum and average Kimura 2-parameter distances) within main notostracan lineages based on COI and 12S rRNA genes.

Species	COI (%)	12S (%)
***T. australiensis***	0.4–14.2 (9.6)	0.0–8.0 (3.6)
***T. cancriformis***	0.0–0.9 (0.4)	0.0–2.3 (0.8)
***T. mauritanicus***	0.9–10.4 (7.0)	0.3–5.1 (2.4)
***T. longicaudatus***	2.2–5.0 (3.9)	0.7
***T. newberryi***	0.2–1.6 (0.8)	-
***T. granarius***	21.1	4.1–11.3 (8.5)
***L. a. apus***	0.2	-
***L.*** ** sp**	0.2–1.6(0.7)	-
***L. viridis***	-	0.6
***Triops***	0.0–30.5 (17.1)	1.0–25.6 (14.7)
***Lepidurus***	14.6–25.9 (16.8)	5.3–10.8 (7.7)

Statistics are only provided for taxa for which multiple sequences were available.

**Figure 2:**
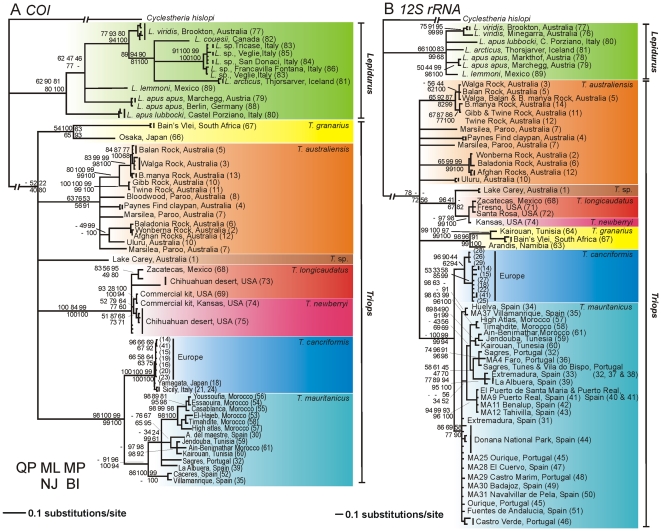
Bayesian inference phylogram based on (A) COI and (B) 12S rRNA sequences. Numbers at nodes represent bootstrap values of quartet puzzling, maximum likelihood, maximum parsimony, neighbour joining and posterior probability of Bayesian inference. Unsupported groupings are indicated using a ‘-’. No value is provided when an alternative placement of the clade in the phylogeny is suggested. Numbers between brackets are locality numbers corresponding to population entries in Table S1.

Overall, phylogenetic analysis of both mitochondrial genes using five different methods of phylogenetic reconstruction resulted in similar topologies (Figure 2) which were confirmed in trees based on combined analysis of the two genes (Figure S1).

The monophyly of *Lepidurus* is confirmed in all trees, except in the QP tree (62%) for the COI gene and in the QP (66%) and BI trees (68%) for the 12S gene. Furthermore, phylogenetic analysis of the unique amino acid sequences translated from the full COI dataset (carried out in PhyML and MrBayes under the MtMam [27] + I model (as selected with the help of ProtTest v.2.4 [28]; results not shown) supported monophyly of the genus.

Phylogenetic reconstruction yielded no statistical support for monophyly of the nominal genus *Triops* nor for an alternative positioning of its lineages. Only ML (52%), NJ (40%) and BI (posterior probability of 80) analyses of the COI gene provide weak support for the monophyly of *Triops*. In the absence of a resolved topology, we resorted to constraint analyses to formally test the hypothesis of monophyly.

Constraint analyses enforcing monophyly of all *Triops* representatives, were conducted for MP and ML trees. Both the Kishino-Hasegawa test and nonparametric Templeton (Wilcoxon signed-ranks) and winning-sites (sign) tests identified the constrained COI and 12S trees, with a length of 1278 and 411 respectively, as significantly more parsimonious (p<0.0001) than the unconstrained MP tree with a length of 1448 and 455 mutational events. When comparing the constrained ML COI and 12S trees (−ln 7250 and 2478 respectively) to the unconstrained ML trees (−ln 7258and 2490 respectively) in Paup*, the Kishino-Hasegawa test significantly (P<0.05) supported the constrained tree as the most likely scenario. K2P distances between the genera also largely exceed those within (Table 1). Based on the whole range of confirmed molecular clocks in invertebrates a (pre) Mesozoic radiation as suggested by fossil remains is highly implausible. According to standard molecular clocks used for crustaceans (1.4–2.8% mya^−1^) initial diversification in the Notostraca started approximately 25.5–12.75 mya. Even according to the slowest molecular clocks, both genera presumably did not diverge before 29.75 mya (based on a COI clock of 1.2% mya^−1^) or before 55.6 mya (based on a 12S clock of 0.5% mya^−1^).

In *Lepidurus*, the basal position of *L. apus lubbocki* is supported by all phylogenetic searches in the COI tree. The Australian *Lepidurus* lineage, which based on morphological traits was traditionally considered a subspecies of *L. apus* (*L. apus viridis*) emerged as a sister species of a clade containing the North American *L. couesii*, *L. arcticus* and the European *Lepidurus* lineages previously identified as *L. couesii*. The monophyly of the subspecies of the presumably widespread *L. apus*, hence, could not be confirmed.

Analyses confirm the monophyly of five main evolutionary lineages within the genus *Triops*: *T. granarius*, *T. cancriformis*, *T. mauritanicus* and a fourth lineage containing *T. longicaudatus* and *T. newberryi*. The fifth lineage comprised haplotypes belonging to a recently discovered *Triops* sp. population from the saline Lake Carey in Western Australia. The monophyly of the various Australian lineages identified as *T. australiensis*, however, could not be confirmed although there was weak support for this clade in the COI dataset. As a result, this taxon could be paraphyletic. Within *Triops*, *T. cancriformis* and *T. mauritanicus* emerged as two sister groups. The minimum genetic distance between these two clades (11.0%) was smaller than the genetic distances to the other main *Triops* lineages (17.9–23.8%). The *Triops* population from Lake Carey in Western Australia did not cluster together with other Australian populations but instead emerged as a distinct lineage. COI and 12S sequences diverged 12.3–17.9% and 7.4–11.1% between haplotypes from Lake Carey and *T. australiensis* specimens, respectively. In the 12S analysis, BI, ML, NJ and QP trees place the American *Triops* clade, which contains specimens morphologically identified as *T. longicaudatus* and *T. newberryi*, as an evolutionary sister of the Australian *T.* sp. clade from Lake Carey. K2P values further justify this position. Maximum genetic divergences between Lake Carey and *T. australiensis* haplotypes of 17.9% and 11.1% in the COI and 12S gene, respectively, were higher than the divergences of 16.3% and 8.6% identified between the Lake Carey species and *T. longicaudatus*.

## Discussion

We reconstructed the first large-scale molecular phylogeny of the primitive crustacean order Notostraca, which is characterised by morphological stasis throughout its fossil record [1], [4], [8], [9]. Based on results from the analysis of two mitochondrial genes (COI and 12S rDNA), we discuss the phylogenetic relationships within this enigmatic group in which morphological taxonomy is complicated by phenotypic variability within and low variability among nominal species.

A preliminary attempt to resolve phylogenetic relations in the Notostraca based on 12S and 16S rDNA markers was performed by Mantovani and coworkers [26]. Splitting the genus *Triops* as suggested by these authors, however, is likely to be unjustified since in our results the monophyly of both genera is confirmed. As a result, the main morphological difference between *Lepidurus* and *Triops*, the presence of a supra-anal plate (a posteriorly directed median extension of the telson which is present in *Lepidurus* but never in *Triops*; Figure 1A, B), is supported as a systematically informative character.

In order to discuss the potential species status of the main notostracan lineages, we will focus mainly on COI, which is the standard marker for barcoding [29]. In branchiopod crustaceans average sequence divergences >7–10% at COI [30], [31], [32] and 4–5% at 12S [33], [34] are typically considered indicative for species level differentiation, although in combination with morphological support, species status has sometimes been attributed to monophyletic clades with lower sequence divergences. Murugan and coworkers [35], for instance, proposed to promote six *T. longicaudatus* lineages to species level although maximum divergence between these lineages observed at the 12S rRNA gene amounted to only 1.6%. In a more recent paper, Korn and coworkers [22] ascribed species status to six morphologically distinguishable *T. mauritanicus* lineages which differed only 2.9–5.1% at the same gene. Depending on the species concept used and without information from hybridization trials confirming reproductive isolation, these decisions can be considered controversial.

### Phylogenetic relations in Lepidurus

Compared to *Triops*, *Lepidurus* has a more restricted distribution. Typical for subarctic and temperate climate zones, the genus is generally replaced by *Triops* in warmer, semi-arid and arid regions. Despite a confirmed presence in the fossil record of the Triassic, there are no reports of current populations in Sub-Saharan Africa [36]. In contrast to the situation in *Triops*, European and North American *Lepidurus* lineages did not emerge as monophyletic groups. This could imply secondary contact between Nearctic and Palearctic *Lepidurus* lineages possibly facilitated by the closer proximity of *Lepidurus* species ranges to northern migration corridors such as the Bering Strait. Efficient dispersal in the (sub) arctic is also suggested by the circumarctic distribution of the recent *L. arcticus* [23]. Birds or mammals (e.g. caribou, moose) which have been shown to be important dispersal vectors of freshwater invertebrates in other regions [37], [38] are likely to have been involved.

Overall, our molecular data suggest that the taxonomy of European and Australian *Lepidurus* lineages is in need of revision. Results confirm the suggestion of Fryer [39] that the Australian *Lepidurus* lineage is not a subspecies of *L. apus* and most likely deserves species status, as the two taxa are paraphyletic and differ 14.7–17.5% at the COI gene. The remaining lineages in *L. apus* (*L. apus lubbocki* and *L. apus apus*), probably also represent different species as they are separated by a genetic distance of 22.1–23.6% and do not form a monophyletic group. According to accepted molecular thresholds, the species status of *L. arcticus* and the European *L.* sp. clade, which was previously considered conspecific to the American *L. couesii*, [3] is confirmed.

### Phylogenetic relations in Triops

Both analyses of a relatively rapid (COI) and a more slowly evolving mitochondrial marker (12SrDNA), consistently recovered a comb-like tree depicting hypothetical phylogenetic relations among the four main *Triops* lineages (*T. granarius*, *T. australiensis*, *T. cancriformis-mauritanicus*, *T. longicaudatus-newberryi*). The possibility of radiation, as suggested for other branchiopod crustaceans [40] and rapid diversification in *Triops* early in its evolutionary history, hence, cannot be excluded. Intercontinental dispersal and subsequent isolation followed by genetic differentiation under limited gene flow almost certainly led to speciation in the four main *Triops* lineages, which are largely restricted to different biogeographic regions. Divergence of the fifth lineage, *T.* sp., in turn, presumably results from a unique habitat shift from freshwater to saline habitats.

Based on molecular clocks, *T. cancriformis* and *T. mauritanicus* most likely diverged between 2.6–12.4 mya confirming the estimate by Korn and coworkers [41] based on 16S rDNA suggesting a potential link with the Messinian Salinity Crisis at the end of the Miocene (5–6 mya). Tectonic activity around the Gibraltar straight, isolating the Mediterranean from the Atlantic Ocean, and low rainfall resulted in strong variation in sea level including near complete drying of the basin [42]. Climate fluctuations, due to loss of the buffering capacity of the Mediterranean, may have led to contraction of suitable *Triops* habitat and a split between *T. mauritanicus* and *T. cancriformis* through vicariance. The clade formed by *T. cancriformis*, which, apart from its mostly European origin, also encompasses a Japanese population, is characterised by a large number of closely related haplotypes. As a result, Mantovani and coworkers [43] concluded that this taxon did not contain cryptic species. Low nucleotide and haplotype diversity over a wide geographical range (Europe and Asia) suggests a relatively recent postglacial colonisation of its current distribution area [25]. A growing number of studies show that postglacially colonised regions are characterised by lower genetic diversity [44], [45]. From the beginning of the Quaternary (2.4 mya) until 10 kya ice sheets cyclically expanded and receded [45]. During cold periods, European *T. cancriformis* populations were most likely restricted to refugia southwards of the ice shelf. In contrast, cryptic diversity was demonstrated in its sister species *T. mauritanicus*, found in Iberia and North Africa [22]. The more southern distribution of this species can explain why it appears to have been less affected by the Pleistocene glaciations than *T. cancriformis* in terms of surviving lineages. Korn and coworkers [22] recognised six morphologically distinguishable lineages (five of which occur in Iberia). As argued by these authors, climate fluctuations in southern Europe associated with the Pleistocene glaciations may have contributed to fragmentation of species ranges facilitating the emergence of different lineages in the Iberian Peninsula through founder effects and genetic drift [46].

Compared to the relatively modest genetic distances in *T. cancriformis* and *T. mauritanicus*, *the T. granarius* clade was shown to harbour more divergent haplotypes. *T. granarius* has a highly scattered distribution including Japan, China [47] and both northern- and southern Africa [48]. Given the vast size of its range it is not surprising that the most distant populations (Japan, southern Africa) are substantially differentiated, with a minimum genetic distance between them of 21.1%. Both COI and 12S datasets suggest that African-Eurasian *T. granarius* consists of different lineages including a South African, Namibian, North African and Japanese clade. Unexpectedly, the two southern African lineages did not cluster together. Instead the South African population was shown to be more closely related to lineages from Tunisia than to Namibian populations (min. K2P distance at 12S of 4.1% vs. 11.3%, respectively). Expanding on the findings by Korn and Hundsdoerfer [48], the South African haplotypes represent a fourth monophyletic lineage in *T. granarius*. Although this is subject to further morphological investigation, genetic distances suggest that the Japanese, the Tunesian, the South African and the Namibian clades probably represent four different species.

The *T. australiensis* clade, in turn, comprises several monophyletic groups and endemic haplotypes exclusive to specific localities. Four clades are restricted to rock pools on granite inselbergs, while the remaining lineages inhabit clay pans. Australian *Triops* are currently grouped into a single species, *T. australiensis* [18], [49] but this may be unjustified since the monophyly of this nominal species is not strongly supported in our analyses. What is more, K2P genetic distances up to 14.2% at the COI gene are well in range of those used by other researchers to distinguish between species in other *Triops* lineages [22], [31], [35]. For example, the clade comprising rock pool populations from Walga Rock, Balan Rock and Bullamanya Rock in Western Australia, minimally diverged 9.4–11.8% at the COI gene from a clay pan population in the same area and 12.0–14.0% from the clade that inhabits the rock pools on the sandstone monolith Uluru in the Northern Territory. The relatively large genetic distances between rock pool and clay pan *Triops* populations in Western Australia contrast with the geographic proximity of these populations providing a firm indication of habitat specialization. Overall, it is clear that *T. australiensis* contains a lot of cryptic diversity. A detailed morphological revision of *T. australiensis* including a discussion of the potential species status of different lineages is currently under preparation (B.V. Timms, unpublished data).

Unexpectedly, the *Triops* sp. population from the saline Lake Carey in Western Australia did not cluster together with other Australian populations, but instead emerged as a distinct lineage. K2P distances between *T.* sp. and its closest relatives *T. australiensis* (min.: 12.3–17.9%) and *T. longicaudatus* (min.: 15.8–16.3%) indicate that this lineage represents a species new to science awaiting formal description (B.V. Timms, in prep.). Tree topologies suggest that the species may have evolved during the initial radiation that gave rise to all present-day lineages coinciding with a unique habitat shift from freshwater to saline systems. Currently, it is the only notostracan population known from saline habitats (105 g L^−1^). Finally, according to 12S tree topologies *T.* sp. could have closer affinities to American than to other Australian *Triops* lineages and may reflect a biogeographic link mediated by historic long distance dispersal. Considering the Cenozoic origin of living Notostraca, this biogeographic link between Australian and American lineages most likely reflects historic long-distance dispersal. Migratory birds and particularly waders, which often feed on branchiopod crustaceans and have been shown to carry propagules, are prime candidate vectors [38]. The bar-tailed godwit subspecies *Limosa lapponica baueri*, for instance, migrates back and forth from Alaska to Australia each year, often in a 11000 km nonstop flight [50] illustrating the potential of long distance dispersal between North America and Australia.

The monophyletic *T. longicaudatus-newberryi* clade is largely endemic to the Americas, while presumed *T. longicaudatus* populations on pacific islands such as the Galápagos, Hawaii and New Caledonia [21], [36] may reflect efficient long distance dispersal, presumably by avian vectors, as discussed above. Japanese records of *T. longicaudatus*, on the other hand, are attributed to recent anthropogenic introductions as a biological control agent in rice fields [48]. Based on our analyses we confirm the monophyly of North American *Triops* populations but not the monophyly of the species *T. newberryi* and *T. longicaudatus*. *T. newberryi* differed only by 0.0–5.2% at COI and 1.0% at 12S from *T. longicaudatus*. The sequenced specimens, hence, should probably be considered conspecific. These findings support the need for a morphological taxonomic revision of *Triops* across North America [51].

### Cryptic diversity and conservation implications

Present-day Triopsidae consist of a limited number of core evolutionary lineages with generally large distributions corresponding to nominal species. However, a complex genetic substructure was shown in certain lineages, such as *T. granarius* and *T. australiensis*, with monophyletic lineages inhabiting different parts of species ranges or contrasting habitat types (e.g. large clay pans versus small ephemeral rock pools). From a conservation point of view, these lineages can be considered evolutionary significant units [52]: appropriate conservation units of which preservation can be recommended. Whether these clades should be raised to species level, despite sometimes modest levels of genetic divergence, is open to discussion and will likely depend on whether reliable diagnostic morphological features can be formally identified.

### Evidence for an ancient radiation?

Although fossils suggest that some living tadpole shrimp species closely resemble fossils as old as 250 million years, both standard and extreme molecular clocks for mitochondrial genes in invertebrates consistently date the most recent common ancestor of all living Triopsidae in the Cenozoic era, with estimates of divergence times among the basal lineages ranging between 29.75 and 55.6 mya (Paleogene period). An ancient (pre) Mesozoic radiation as suggested by fossil remains, on the other hand, would explain today's distribution of lineages by vicariance rather than long distance dispersal of several lineages. If we would assume that *Lepidurus* and *Triops* indeed existed as separate lineages in the middle Triassic (220 mya) then this would imply a mutation rate at the COI gene of about 0.16% per mya which is highly unrealistic since the lowest rate of evolution observed at this gene in invertebrates is 1.2% per mya [53]. Contemporary tadpole shrimp species thus almost certainly are the result of a more recent radiation from a single ancestral lineage surviving into the Tertiary rather than a group of relict lineages from an earlier (pre-) Mesozoic radiation that presumably gave rise to a number of extinct ancient lineages known from the fossil record [4]. The scenario of a recent radiation, dispersal and speciation in isolation adequately explains why, despite a (pre-) Pangaean origin of the Triopsidae, a number of lineages are linked to biogeographic regions (e.g. *T. australiensis* and the Australian *Lepidurus* sp. in Australia, *T. longicaudatus-newberryi* in the Americas, and *T. cancriformis* in the Palearctic).

The supra-anal plate, which is the key diagnostic character to distinguish *Lepidurus* from *Triops*, is a trait which modern *Lepidurus* species share with a number of Triassic and Cretaceous fossils [11], [12], [36]. Given the recent origin of *Lepidurus*, Mesozoic tadpole shrimp with supra-anal plates probably should not be classified in the same genus. The supra-anal plate, as such, can be a primitive character which has been lost both in a number of fossil Triopsidae as well as in the extant *Triops* representatives. On the other hand, considering the fact that the oldest known triopsid fossils lack a supra-anal plate [5], it is also possible that it is a derived trait which has evolved multiple times both in Mesozoic triopsids and, again, in the common ancestor of modern *Lepidurus* lineages. Evidently, current tadpole shrimp species having evolved quite recently are not living fossils and the myth that *T. cancriformis* would be the oldest species on the planet must be firmly discredited. “Living fossil" is undoubtedly an attractive tag to draw attention to peculiar taxa exhibiting primitive traits. Yet, this term can be misleading and the intrinsic scientific value of such a label is not uncontested. Different definitions are in use and particularly in popular scientific literature “living fossil" is often used over-simplistically as a term to designate an ancient species which has presumably survived relatively unchanged until present day. Not surprisingly, creationist lobbyists eagerly enumerate examples of morphological stasis [54] although these by no means provide evidence against evolution by natural selection. Nonetheless, the “living fossil" concept, which was originally coined by Darwin [55], can also be more stringently and realistically defined as a taxon which belongs to a group with a long evolutionary history, has retained a number of primitive characters and has few living relatives. According to this definition the members of the order Notostraca, in general, can be considered living fossils. At least two main factors are likely to have contributed to morphological stasis in tadpole shrimp: the simple body plan consisting of a dorsal armor and serially repeated structures; traits which are also present in other “living fossils" such as horseshoe crabs [56] and chitons [57], and the very specific habitat type in which these organisms have persisted during their evolutionary history. Since the appearance of planktivorous fish in the Devonian and Carboniferous, large predation sensitive branchiopod crustaceans such as Notostraca are restricted to extreme aquatic systems that lack fish such as temporary ponds and saline lakes: a very specific niche [4], [58] in which they still persist today.

### Conclusions

Although some present day tadpole shrimp species closely resemble fossil specimens as old as 250 mya, no molecular support was found for an ancient (pre) Mesozoic radiation. Instead, living tadpole shrimp are most likely the result of a relatively recent radiation in Cenozoic and close resemblances between recent and fossil taxa are probably the result of the highly conserved general morphology in this group and of homoplasy. It is clear that more and more evidence is accumulating indicating that a lack of readily observable phenotypic change (morphological stasis) during the evolutionary history of a certain lineage does not necessarily imply evolutionary stasis [59]. As shown in this study, recent species which are virtually identical to fossils in terms of their morphology may represent very different evolutionary lineages.

## Methods

COI and 12S rRNA genes were sequenced for up to six tadpole shrimp specimens per population. DNA extraction, polymerase chain reaction and sequencing protocols are provided in Text S2. All new samples were collected by the authors in the field between 2008 and 2010, using a simple dipnet (5 mm mesh). Exceptionally, *T. newberryi* specimens from a population in Kansas, USA were laboratory-hatched from sediment in distilled water at 20°C.

### Ethics statement

Collected animals were anaesthetized in carbonized water before transfer to ethanol. Collection and export permits were granted by the Free State Province Department of Tourism, Environmental and Economic affairs (South Africa): permit no.: HK/P1/07375/001 and by the Australian government: permit no. SF007548 and SF005789.

### Genetic data analyses

Sequences were aligned (ClustalW multiple alignment: [60]) and trimmed in BioEdit Sequence Alignment Editor v.7.0.0 [61]. 120 additional COI and 74 12S sequences were drawn from GenBank and aligned to the newly obtained DNA fragments (Table S1 provides additional details and GenBank accession codes). The cyclestherid conchostracan *Cyclestheria hislopi* (Baird, 1859) was selected as outgroup. Finally the alignment was inspected by eye for any anomalies and found to be straightforward. All new sequences were deposited in GenBank under accession codes (JN175223–267; JN190396–398).

For the COI and 12S datasets, jModeltest v.0.1.1 [62] respectively selected the TIM1+I+G (with a proportion of invariable sites of 0.496 and a gamma-shape parameter of 0.775) model, with nucleotide frequencies A = 0.32, C = 0.18, G = 0.10 and T = 0.38 and rate matrix (1.00, 23.28, 2.06, 2.06, 14.10, 1.00) and the TPM2uf+G (with a gamma-shape parameter of 0.33) model, with nucleotide frequencies A = 0.37, C = 0.17, G = 0.11 and T = 0.35 and rate matrix (11.35, 46.47, 11.35, 1.00, 46.47, 1.00) as best fitting models of evolution. Model averaged phylogeny analyses were performed in the same software, indicating that all 88 tested models rendered nearly identical trees for both the COI and 12S data.

Dating splits between passively dispersed aquatic invertebrates is problematic since long distance and even intercontinental dispersal mediated by vectors such as water birds is a realistic possibility [63], [64]. In addition, the highly conserved general morphology in Notostraca throughout their evolutionary history impedes the use of fossils to calibrate molecular clocks. A likelihood ratio test [65] performed in TREE-PUZZLE [66] rejected clock-like evolution for both the COI and 12S datasets.

Even though this means that we cannot linearly calculate divergence times for individual splits in the phylogenetic trees based on genetic distance, we can broadly estimate the timing of diversification and the likeliness of an ancient radiation by using the range of molecular clocks known for invertebrates. Although this approach which is used due to the impossibility of fossil calibration is relatively coarse, at the very least it allows distinguishing between an ancient (pre) Mesozoic radiation suggested by fossil remains and a more recent Tertiary or Quaternary radiation. A prerequisite, however, is that sequences are not oversaturated in terms of accumulated mutations. As a result, substitution saturation for the third codon position was tested for both the COI data in DAMBE 5.2.13 [67]. The index of substitution saturation (Iss) was found to be significantly smaller than the critical index of substitution saturation (Iss c), indicating little saturation. Generally accepted COI evolution rates for arthropods are in the range of 1.40–2.6% mya^−1^ [68]–[69]. Slowest and fastest rates of COI evolution in invertebrates are reported in bathysciine beetles (1.2% mya^−1^; [53]) and barnacles (4.9% mya^−1^; [70]), respectively. For the 12S rRNA coding region, we apply an evolutionary rate of 0.5% mya^−1^ [71] which is commonly used in branchiopod crustaceans [72].

Genetic distances were computed in MEGA v.4.1. [73] using Kimura 2-parameter (K2P) distances [74] allowing for comparison with earlier studies. Between haplotype, within and between species and within and between genus divergences were calculated.

Phylogenetic reconstruction was performed for both mitochondrial DNA datasets independently, using neighbor joining (NJ), maximum parsimony (MP), maximum likelihood (ML), quartet puzzling (QP) methods and Bayesian inference (BI). MP analyses were conducted in Paup* v.4.0b10 [75] using the PaupUp graphical interface [76]. For the ML analyses PhyML [77] was used. ML analyses in PhyML (1000 bootstrap replicates, NNI) were run according to the evolutionary model and parameters as selected by jModeltest. NJ analyses were performed in MEGA using the following settings: maximum composite likelihood, Tamura-Nei substitution model, defined G and 1000 bootstrap replicates. Quartet puzzling maximum likelihood analyses were performed in TREE-PUZZLE according to the model and parameters selected by jModeltest. Bayesian analyses were conducted in MrBayes v.3.1.2 [78] according to the evolutionary model and parameters suggested by jModeltest. MrBayes ran for 5×10^6^ generations (lset number of substitution types = 6, rates = invgamma, number of rate categories for the gamma distribution = 4, sampling frequency = 100 generations) until a standard deviation of split frequencies of 0.0078 was attained. An outgroup (*C. hislopi*) was defined and in order to only include trees in which convergence of the Markov chain had been reached, we chose a burn-in of 25%. The remaining trees were used to construct a 50% majority consensus tree.

Finally, in order to integrate the information provided by both genes, phylogenetic analyses were also conducted on a combined dataset containing both COI and 12S sequences. Parameters for both genes were estimated independently in MrBayes using the ‘unlink’ command (partition twogenes = 2: 12S, COI, lset applyto = (1), nst = 6, rates = invgamma, ngammacat = 4, lset applyto = (2), nst = 6, rates = invgamma, ngammacat = 4, unlink shape = all). MrBayes ran for 8×10^6^ generations with a sampling frequency of 100 and a defined outgroup (*C. hislopi*).

In case phylogenetic analyses did not unequivocally support monophyly of the two Notostracan genera, constraint analysis using Kishino-Hasegawa- [79] and Shimodaira-Hasegawa [80] tests for the ML tree and Kishino-Hasegawa as well as Templeton - and winning site tests for the MP tree were conducted in Paup* to test whether enforcing monophyly of genera led to a statistically significant increase in tree likelihood.

## Supporting Information

Figure S1 Bayesian inference phylogram based on combined COI and 12s rRNA sequences. Numbers at nodes represent bootstrap values of maximum likelihood (ML), maximum parsimony (MP) and posterior probability values of Bayesian inference (BI). Unsupported groupings are indicated using a ‘-’. No value is provided if this method of phylogenetic inference would suggest an alternative placement of the corresponding clade in the phylogeny.
(DOCX)

Table S1 Overview of investigated Notostraca samples.
(DOC)

Table S2 Kimura 2-parameter distance matrix (min.-max.) between investigated notostracan lineages based on COI (below diagonal) and 12S rRNA (above diagonal) genes. Empty cells indicate that sequence information was unavailable.
(DOCX)

Table S3 Divergence times between main Triops clades (minimum-maximum) based on the standardly used average COI molecular clock (1.40% mya^−1^; below diagonal) and 12S molecular clock (0.5% mya^−1^; above diagonal) for crustaceans.
(DOCX)

Table S4 Uncorrected p distance matrix (min.-max.) between investigated notostracan lineages based on COI (below diagonal) and 12S rRNA (above diagonal) genes. Empty cells indicate that sequence information was unavailable.
(DOCX)

Text S1 Overview of currently accepted Notostraca species and their known distributions.
(DOC)

Text S2 DNA isolation, molecular markers, polymerase chain reaction and sequencing.
(DOC)
