# Supplementary material for: Toward a Global Phylogeny of the “Living Fossil" Crustacean Order of the Notostraca
Source: PLoS One. 2012 Apr 18;7(4):e34998. doi: 10.1371/journal.pone.0034998 (PMC3329532; doi:10.1371/journal.pone.0034998)

***Figure S1.*** *Bayesian inference phylogram based on combined COI and 12s rRNA sequences. Numbers at nodes represent bootstrap values of maximum likelihood (ML), maximum parsimony (MP) and posterior probability values of Bayesian inference (BI).Unsupported groupings are indicated using a ‘-‘. No value is provided if this method of phylogenetic inference would suggest an alternative placement of the corresponding clade in the phylogeny.*


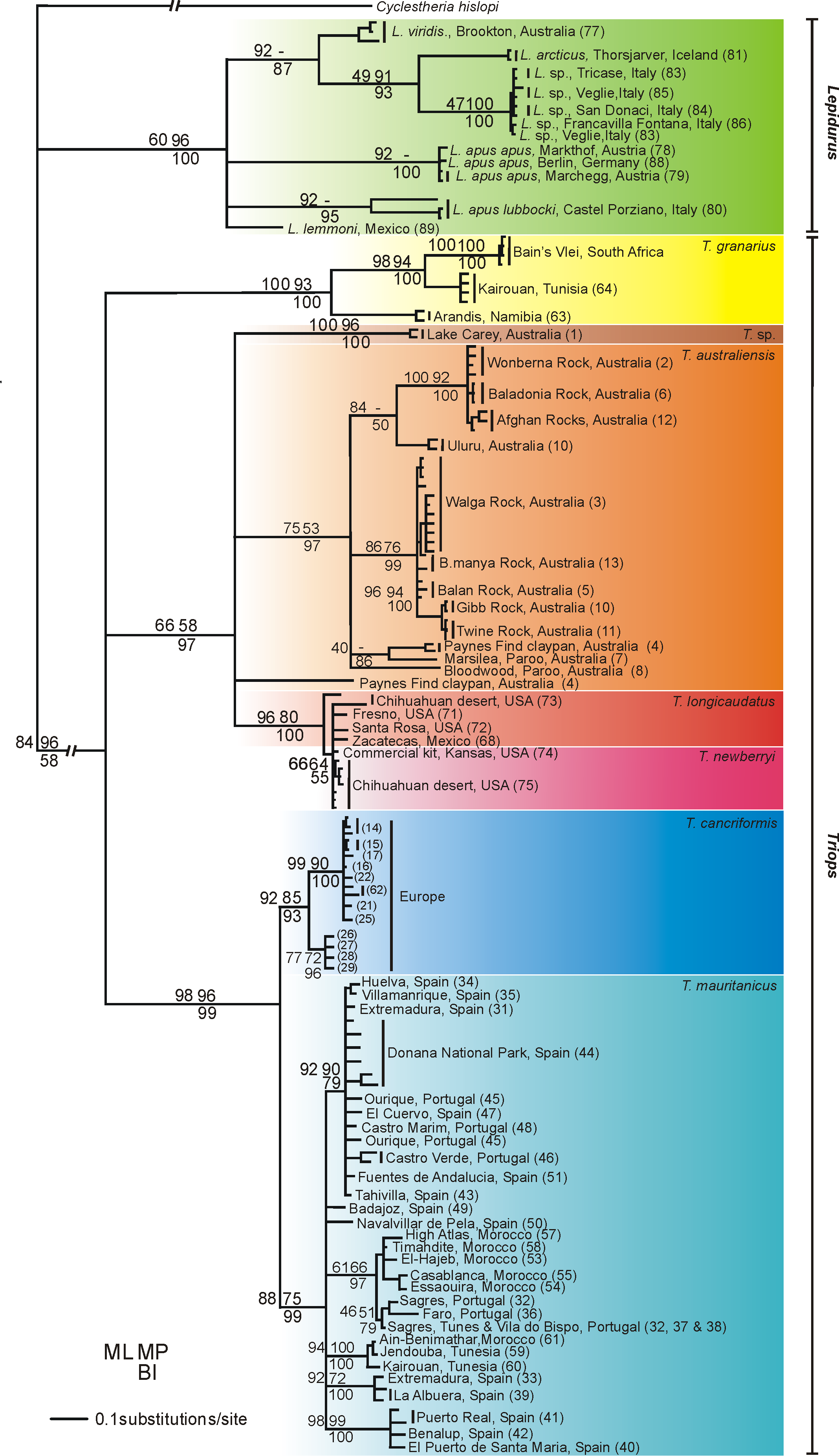

Supplement: Figure S1 — Bayesian inference phylogram based on combined COI and 12s rRNA sequences. Numbers at nodes represent bootstrap values of maximum likelihood (ML), maximum parsimony (MP) and posterior probability values of Bayesian inference (BI). Unsupported groupings are indicated using a ‘-’. No value is provided if this method of phylogenetic inference would suggest an alternative placement of the corresponding clade in the phylogeny. (DOCX) [file pone.0034998.s001.docx]
