# Supplementary material for: Toward a Global Phylogeny of the “Living Fossil" Crustacean Order of the Notostraca
Source: PLoS One. 2012 Apr 18;7(4):e34998. doi: 10.1371/journal.pone.0034998 (PMC3329532; doi:10.1371/journal.pone.0034998)
Supplement: Table S1 — Overview of investigated Notostraca samples. (DOC) [file pone.0034998.s002.doc]

**Table S1**: *Overview of investigated Notostraca samples*

| **Pop.** | **Species** | **Locality** | **Latitude** | | | | **Longitude** | | | | **12S** | **COI** |
| --- | --- | --- | --- | --- | --- | --- | --- | --- | --- | --- | --- | --- |
| **1** | *T.* sp. | Lake Carey, WA, Australia | S | 29 | 05 | 04 | E | 122 | 18 | 56 | JN175250-1 | JN175235-6 |
| **2** | *T. australiensis* | Wonberna Rock, WA, Australia | S | 32 | 35 | 00 | E | 123 | 46 | 00 | JN175252 | JN175237 |
| **3** | *T. australiensis* | Walga Rock, WA, Australia | S | 27 | 24 | 16 | E | 117 | 27 | 46 | JN175253 | JN175238-40 |
| **4** | *T. australiensis* | Claypan SE of Paynes Find, WA, Australia | S | 29 | 19 | 53 | E | 117 | 47 | 12 | JN175254-5 | JN175242 |
| **5** | *T. australiensis* | Balan Rock, WA, Australia | S | 28 | 10 | 51 | E | 117 | 25 | 32 | JN175258 | JN175230-2 |
| **6** | *T. australiensis* | Baladonia Rock, WA, Australia | S | 32 | 28 | 00 | E | 123 | 52 | 00 | JN175259 | JN175233 |
| **7** | *T. australiensis* | Marsilea pool (Middle Paroo), NSW, Australia | S | 29 | 32 | 13 | E | 144 | 52 | 26 | DQ343233.1-2.1 | DQ343234-35 |
| **8** | *T. australiensis* | Paroo area, NSW, Bloodwood station, Australia | S | 29 | 32 | 13 | E | 144 | 52 | 26 |  | EF189677.1 |
| **9** | *T. australiensis* | Uluru (Ayers Rock), NT,Australia | S | 25 | 20 | 42 | E | 131 | 01 | 57 | JN175263 | JN175245 |
| **10** | *T. australiensis* | Gibb Rock, WA,Australia | S | 32 | 06 | 60 | E | 119 | 01 | 02 | JN175265 | JN190396 |
| **11** | *T. australiensis* | Twine Rock, WA,Australia | S | 32 | 06 | 44 | E | 118 | 57 | 15 | JN175267 | JN190398x |
| **12** | *T. australiensis* | Afghan Rocks, WA, Australia | S | 32 | 21 | 18 | E | 123 | 40 | 22 | JN175244 | JN175264 |
| **13** | *T. australiensis* | Bullamanya Rock, WA, Australia | S | 29 | 19 | 53 | E | 117 | 47 | 12 | JN175261 | JN190397 |
| **14** | *T. cancriformis cancriformis* | Brasschaat, Belgium | N | 51 | 17 | 23 | E | 04 | 29 | 30 | JN175256-257 | JN175234 |
| **15** | *T. cancriformis cancriformis* | Camargue, France | N | 43 | 40 | 39 | E | 04 | 22 | 08 | JN175260 | JN175241 |
| **16** | *T. cancriformis cancriformis* | Tiszabercelgi, Hungary | N | 48 | 09 | 22 | E | 21 | 38 | 46 | AM184167.1 | EF675849.1 |
| **17** | *T. cancriformis cancriformis* | Morava, Austria | N | 48 | 44 | 45 | E | 16 | 54 | 20 | AF494482.1 | EF189678.1 |
| **18** | *T. cancriformis cancriformis* | Yamagata prefecture, Japan | N | 40 | 45 | 28 | E | 141 | 14 | 28 |  | EF675828.1 |
| **19** | *T. cancriformis cancriformis* | Königswartha, Germany | N | 51 | 18 | 36 | E | 14 | 19 | 33 |  | EF675833.1 |
| **20** | *T. cancriformis cancriformis* | Morava Flood Plain, Czech republic | N | 49 | 57 | 22 | E | 16 | 11 | 58 |  | EF675850.1 |
| **21** | *T. cancriformis cancriformis* | Gorgo di Baglio, Italy (Sicily) | N | 37 | 57 | 50 | E | 12 | 46 | 15 |  | EF675881.1 |
| **22** | *T. cancriformis cancriformis* | Melenci, pond 2, Serbia | N | 45 | 30 | 60 | E | 20 | 18 | 60 | AM184170.1 |  |
| **23** | *T. cancriformis cancriformis* | Godshill pond, United Kingdom | N | 50 | 37 | 59 | W | 01 | 15 | 16 |  | EF675858.1 |
| **24** | *T. cancriformis cancriformis* | Italy (Sicily) |  |  |  |  |  |  |  |  |  | DQ369316.1 |
| **25** | *T. cancriformis cancriformis* | Principina a Mare, Grosseto, Italy | N | 42 | 45 | 44 | E | 11 | 06 | 48 | AY159564-5.1 |  |
| **26** | *T. cancriformis cancriformis* | Vienna, Austria | N | 48 | 12 | 28 | E | 16 | 22 | 28 | AM269414.1 |  |
| **27** | *T. cancriformis cancriformis* | Morava Flood Plain, Austria | N | 48 | 44 | 45 | E | 16 | 54 | 20 | AF494482.1 |  |
| **28** | *T. cancriformis cancriformis* | Austria, commercial kit |  |  |  |  |  |  |  |  | AM184166.1 |  |
| **29** | *T. cancriformis cancriformis* | Uljanowsk, Russia | N | 54 | 19 | 10 | E | 48 | 23 | 06 | AM184169.1 |  |
| **30** | *T. mauritanicus* | Ares del Maestre, Spain | N | 40 | 27 | 25 | W | 00 | 08 | 01 |  | EF675907.1 |
| **31** | *T. mauritanicus* | Extremadura (4 pools), Spain | N | 39 | 28 | 00 | W | 05 | 52 | 60 | AM184184.1 |  |
| **32** | *T. mauritanicus* | Sagres, Portugal | N | 36 | 60 | 24 | W | 08 | 56 | 39 | AM184181.1  FN691426.1 | FN691444.1 |
| **33** | *T. mauritanicus* | Extremadura, pond Gitanilla, Spain | N | 39 | 28 | 00 | W | 05 | 52 | 60 | AM184182.1 |  |
| **34** | *T. mauritanicus* | Huelva, Spain | N | 37 | 15 | 24 | W | 06 | 56 | 57 | AM184183.1 |  |
| **35** | *T. mauritanicus* | Villamanrique, Spain | N | 37 | 15 | 10 | W | 06 | 19 | 05 | FN691392.1  FN691395.1 | FN691434.1 |
| **36** | *T. mauritanicus* | Faro, Portugal | N | 37 | 01 | 05 | W | 07 | 56 | 09 | FN691425.1 |  |
| **37** | *T. mauritanicus* | Tunes, Portugal | N | 37 | 10 | 00 | W | 08 | 15 | 36 | FN691423-4.1 |  |
| **38** | *T. mauritanicus* | Vila do Bispo, Portugal | N | 37 | 05 | 01 | W | 08 | 54 | 36 | FN691422.1 |  |
| **39** | *T. mauritanicus* | La Albuera, Spain | N | 38 | 43 | 24 | W | 06 | 49 | 56 | FN691427-8.1 | FN691435.1 |
| **40** | *T. mauritanicus* | El Puerto de Santa Maria, Spain | N | 36 | 35 | 28 | W | 06 | 14 | 10 | FN691421.1 |  |
| **41** | *T. mauritanicus* | Puerto Real, Spain | N | 36 | 31 | 37 | W | 06 | 11 | 40 | FN691419-20.1 |  |
| **42** | *T. mauritanicus* | Benalup, Spain | N | 36 | 20 | 56 | W | 05 | 48 | 13 | FN691418.1 |  |
| **43** | *T. mauritanicus* | Tahivilla, Spain | N | 36 | 11 | 14 | W | 05 | 45 | 18 | FN691417.1 |  |
| **44** | *T. mauritanicus* | Donana National Park, Spain | N | 36 | 58 | 11 | W | 06 | 21 | 42 | FN691389-91.1  FN691397.1  FN691402-3.1  FN691414-6.1  FN691409-10.1  FN691407.1  FN691411-2.1  FN691408.1  FN691405-6.1 |  |
| **45** | *T. mauritanicus* | Ourique, Portugal | N | 37 | 39 | 11 | W | 08 | 13 | 36 | FN691404.1  FN691396.1 |  |
| **46** | *T. mauritanicus* | Castro Verde, Portugal | N | 37 | 43 | 00 | W | 08 | 05 | 17 | FN691393-4.1 |  |
| **47** | *T. mauritanicus* | El Cuervo, Spain | N | 36 | 51 | 07 | W | 06 | 02 | 27 | FN691401.1 |  |
| **48** | *T. mauritanicus* | Castro Marim, Portugal | N | 37 | 13 | 41 | W | 07 | 27 | 03 | FN691400.1 |  |
| **49** | *T. mauritanicus* | Badajoz, Spain | N | 38 | 52 | 79 | W | 06 | 58 | 26 | FN691399.1 |  |
| **50** | *T. mauritanicus* | Navalvillar de Pela, Spain | N | 39 | 05 | 23 | W | 05 | 28 | 06 | FN691398.1 |  |
| **51** | *T. mauritanicus* | Fuentes de Andalucia, Spain | N | 37 | 27 | 39 | W | 05 | 21 | 11 | FN691413.1 |  |
| **52** | *T. mauritanicus* | Caceres, Spain | N | 39 | 28 | 47 | W | 06 | 22 | 18 |  | FN691433.1 |
| **53** | *T. mauritanicus* | El-Hajeb, Morocco | N | 33 | 41 | 53 | W | 05 | 21 | 52 |  | FN691443.1 |
| **54** | *T. mauritanicus* | Essaouira, Morocco | N | 31 | 31 | 32 | W | 09 | 47 | 10 |  | FN691440.1 |
| **55** | *T. mauritanicus* | Casablanca, Morocco | N | 33 | 36 | 29 | W | 07 | 38 | 02 |  | FN691439.1 |
| **56** | *T. mauritanicus* | Youssoufia, Morocco | N | 32 | 14 | 41 | W | 08 | 31 | 26 |  | EF675905.1 |
| **57** | *T. mauritanicus* | High Atlas S. of Marrakech, Morocco | N | 31 | 19 | 38 | W | 07 | 58 | 54 | AM184180.1 | FN691441.1 |
| **58** | *T. mauritanicus* | Timahdite, Morocco | N | 33 | 32 | 02 | W | 05 | 06 | 11 | AM184176.1 | FN691442.1 |
| **59** | *T. mauritanicus* | Jendouba, Tunisia | N | 36 | 30 | 02 | E | 08 | 47 | 01 | AM184173.1 | FN691437.1 |
| **60** | *T. mauritanicus* | Kairouan, Tunisia | N | 35 | 40 | 33 | E | 10 | 05 | 30 | AM184172.1 | FN691436.1 |
| **61** | *T. mauritanicus* | Ain-Benimathar T.c.s. M., Morocco | N | 34 | 29 | 42 | W | 02 | 01 | 55 | AM184174.1 | FN691438.1 |
| **62** | *T. cancriformis* | Espolla, Spain | N | 42 | 23 | 22 | E | 03 | 00 | 05 | DQ369307-8.1 | DQ369317.1 |
| **63** | *T. granarius* | Arandis, Namibia | S | 22 | 24 | 27 | E | 14 | 58 | 24 | AM269422-23.1 |  |
| **64** | *T. granarius* | Kairouan, Tunisia | N | 35 | 40 | 33 | E | 10 | 05 | 30 | AM269416-17.1 |  |
| **65** | *T. granarius* | Hajib Al´Uyun, Tunisia | N | 35 | 23 | 47 | E | 09 | 32 | 34 | AM269419-20.1 |  |
| **66** | *T. granarius* | Osaka, Japan | N | 34 | 41 | 36 | E | 135 | 30 | 64 |  | GQ144446.1 |
| **67** | *T. granarius* | Bain's Vlei, South Africa | S | 29 | 02 | 60 | E | 26 | 07 | 00 | JN175248-9 | JN175223-4 |
| **68** | *T. longicaudatus* | Zacatecas, Mexico |  |  |  |  |  |  |  |  |  | GQ144444.1 |
| **69** | *T. longicaudatus* | *Triops*world commercial kit, USA |  |  |  |  |  |  |  |  |  | AY639934.1 |
| **70** | *T. longicaudatus* | Fresnillo-Cuencame, Zacatecas, Mexico | N | 23 | 32 | 46 | W | 102 | 57 | 34 | AY115600.1 |  |
| **71** | *T. longicaudatus* | Fresno, USA | N | 36 | 44 | 50 | W | 119 | 46 | 22 | AY159566.1 |  |
| **72** | *T. longicaudatus* | Santa Rosa, USA | N | 38 | 26 | 20 | W | 122 | 42 | 58 | AJ000817.1 |  |
| **73** | *T. longicaudatus* | Chihuahuan desert, USA | N | 32 | 30 | 25 | W | 106 | 44 | 50 |  | HQ908517-67.1 |
| **74** | *T. newberryi* | Kansas, USA, Commercial kit | N | 39 | 01 | 24 | W | 98 | 27 | 35 | JN175262 | JN175243 |
| **75** | *T. newberryi* | Chihuahuan desert, USA | N | 32 | 30 | 25 | W | 106 | 44 | 50 |  | HQ908496-516.1 |
| **76** | *L. viridis* | Minegarra pool, Australia | S | 30 | 46 | 09 | E | 115 | 32 | 43 | JN175266 |  |
| **77** | *L. viridis.* | Brookton, Australia | S | 32 | 22 | 30 | E | 117 | 00 | 35 | JN175225-9 | JN175225-6x |
| **78** | *L. apus apus* | Markthof, Austria | N | 48 | 11 | 37 | E | 16 | 57 | 26 | AY1-59568 | DQ834543.1 |
| **79** | *L. apus apus* | Marchegg, Austria | N | 48 | 15 | 45 | E | 16 | 54 | 40 |  | DQ834544.1  DQ148285.1 |
| **80** | *L. apus lubbocki* | Castel Porziano, Italy | N | 41 | 44 | 38 | E | 12 | 24 | 11 | AY159567  DQ148273.1 | DQ834540-2.1  DQ148282-4.1 |
| **81** | *L. arcticus* | Thjorsarver, Iceland | N | 64 | 48 | 47 | W | 18 | 48 | 38 | AY159569 | DQ834545.1  DQ148286.1 |
| **82** | *L. couesii* | Canada |  |  |  |  |  |  |  |  |  | DQ310622 |
| **83** | *L.* sp. | Tricase, Italy | N | 39 | 55 | 60 | E | 18 | 21 | 27 |  | DQ834549-50.1 |
| **84** | *L.* sp. | San Donaci, Italy | N | 40 | 27 | 07 | E | 17 | 55 | 30 |  | DQ834548.1  DQ148289.1 |
| **85** | *L.* sp. | Veglie, Italy | N | 40 | 20 | 18 | E | 17 | 57 | 44 |  | DQ834546-7.1  DQ148288.1 |
| **86** | *L.* sp. | Francavilla Fontana, Italy | N | 40 | 32 | 36 | E | 17 | 34 | 58 |  | DQ148290.1 |
| **87** | *L.* sp. | Manduria, Italy | N | 40 | 24 | 05 | E | 17 | 38 | 04 |  | DQ148287.1 |
| **88** | *L. apus apus* | Berlin, Germany | N | 52 | 31 | 9 | E | 13 | 24 | 50 |  | EF189669.1 |
| **89** | *L. lemmoni* | Mexico |  |  |  |  |  |  |  |  | AY115604.1 | GQ144447.1 |
