# Supplementary material for: Toward a Global Phylogeny of the “Living Fossil" Crustacean Order of the Notostraca
Source: PLoS One. 2012 Apr 18;7(4):e34998. doi: 10.1371/journal.pone.0034998 (PMC3329532; doi:10.1371/journal.pone.0034998)
Supplement: Table S2 — Kimura 2-parameter distance matrix (min.-max.) between investigated notostracan lineages based on COI (below diagonal) and 12S rRNA (above diagonal) genes. Empty cells indicate that sequence information was unavailable. (DOCX) [file pone.0034998.s003.docx]

**Table S2.** *Kimura 2-parameter distance matrix (min.-max.) between investigated notostracan lineages based on COI (below diagonal) and 12S rRNA (above diagonal) genes. Empty cells indicate that sequence information was unavailable*

|  | **1** | **2** | **3** | **4** | **5** | **6** | **7** | **8** | **9** | **10** | **11** | **12** | **13** | **14** |
| --- | --- | --- | --- | --- | --- | --- | --- | --- | --- | --- | --- | --- | --- | --- |
| **1.** *T. australiensis* | - | 7.4-11.1 | 12.4-19.2 | 11.5-18.4 | 7.4-10.6 | 6.1-11.1 | 10.5-20.1 | 16.5-20.2 | 16.6-19.0 | 19.1-21.1 | - | - | 13.9-16.6 | 17.2-21.0 |
| **2.** *T.* sp. | 12.3-17.9 | - | 14.3-15.9 | 12.9-16.3 | 7.7-8.6 | 7.7 | 16.1-20.9 | 19.6 | 18.0 | 19.9 | - | - | 16.2 | 20.4 |
| **3.** *T. cancriformis* | 17.9-22.8 | 21.6-21.9 | - | 1.3-6.2 | 13.7-16.6 | 13.2-14.7 | 21.9-27.8 | 19.8-21.2 | 18.0-19.2 | 21.9-23.1 | - | - | 17.4-19.8 | 18.7-19.9 |
| **4.** *T. mauritanicus* | 18.8-27.5 | 22.0-25.3 | 11.0-14.8 | - | 11.0-15.7 | 11.3-14.4 | 19.6-26.9 | 22.2-26.5 | 19.0-22.7 | 21.5-25.9 | - | - | 18.3-22.1 | 20.9-25.1 |
| **5.** *T. longicaudatus* | 13.3-20.3 | 15.8-16.3 | 18.1-21.6 | 17.9-24.0 | - | 1.0 | 15.4-19.7 | 20.4-21.1 | 18.2-18.8 | 20.7-21.3 | - | - | 18.1-18.8 | 19.9-20.6 |
| **6.** *T. newberryi* | 13.7-19.7 | 15.8-17.4 | 20.2-21.4 | 19.5-23.7 | 0.0-5.2 | - | 16.4-20.0 | 19.1 | 17.4 | 19.8 | - | - | 16.9 | 19.2 |
| **7.** *T. granarius* | 17.5-27.6 | 17.0-26.9 | 28.2-30.5 | 23.8-30.4 | 20.5-24.9 | 21.7-24.3 | - | 20.9-24.1 | 21.0-27.0 | 22.9-26.3 | - | - | 16.8-20.9 | 23.5-26.4 |
| **8.** *L.viridis* | 24.0-29.0 | 25.2-26.5 | 26.8-27.7 | 25.9-30.0 | 26.1-29.5 | 27.4-30.2 | 20.4-26.7 | - | 8.5 | 10.7 | - | - | 7.6 | 6.2 |
| **9.** *L. a. apus* | 20.8-31.7 | 23.7-24.3 | 23.3-24.2 | 26.0-31.4 | 23.6-27.5 | 25.2-26.8 | 26.2-29.0 | 17.9-18.7 | - | 8.0 | - | - | 5.2 | 5.2 |
| **10.** *L. a. lubbocki* | 22.5-29.0 | 26.0-26.7 | 23.8-24.8 | 24.1-28.0 | 23.0-25.0 | 23.6-25.7 | 26.3-27.6 | 21.4-22.8 | 22.1-23.6 | - | - | - | 10.3 | 11.1 |
| **11.** *L. couesii* | 27.6-33.1 | 31.4-31.8 | 23.7 | 27.1-30.0 | 26.1-28.9 | 27.5-28.9 | 28.9-29.1 | 14.7-15.4 | 21.9-22.4 | 21.6-22.1 | - | - | - | - |
| **12.** *L.* sp. | 27.2-35.7 | 30.1-31.6 | 28.0-28.9 | 28.8-33.3 | 27.0-30.5 | 27.9-31.2 | 28.0-31.1 | 15.9-17.5 | 22.5-24.3 | 24.2-26.2 | 10.8-11.3 | - | - | - |
| **13.** *L. lemmoni* | 23.5-27.9 | 22.5 | 25.2-25.5 | 24.2-28.2 | 24.4-26.3 | 25.3-26.0 | 24.7-26.9 | 16.6-17.1 | 21.4-21.9 | 22.6-23.3 | 21.9 | 23.1-23.8 | - | 7.7 |
| **14.** *L. arcticus* | 26.9-31.1 | 28.4-29.2 | 28.1-28.8 | 27.7-31.5 | 26.2-28.9 | 27.4-29.5 | 29.6-30.7 | 18.0-19.1 | 23.0-23.9 | 20.8-21.7 | 15.5-15.8 | 14.3-15.4 | 22.0-22.3 | - |
