# Supplementary material for: Toward a Global Phylogeny of the “Living Fossil" Crustacean Order of the Notostraca
Source: PLoS One. 2012 Apr 18;7(4):e34998. doi: 10.1371/journal.pone.0034998 (PMC3329532; doi:10.1371/journal.pone.0034998)
Supplement: Table S3 — Divergence times between main Triops clades (minimum-maximum) based on the standardly used average COI molecular clock (1.40% mya−1; below diagonal) and 12S molecular clock (0.5% mya−1; above diagonal) for crustaceans. (DOCX) [file pone.0034998.s004.docx]

**Table S3*.*** *Divergence times between main Triops clades (minimum-maximum) based on the standardly used average COI molecular clock (1.40% mya^-1^; below diagonal) and 12S molecular clock (0.5% mya^-1^; above diagonal) for crustaceans.*

|  | **1** | **2** | **3** | **4** | **5** | **6** | **7** | **8** | **9** | **10** | **11** | **12** | **13** | **14** |
| --- | --- | --- | --- | --- | --- | --- | --- | --- | --- | --- | --- | --- | --- | --- |
| **1.** *T. australiensis* | - | 14.8-22.2 | 24.8-38.4 | 23.0-36.8 | 14.8-21.2 | 12.2-22.2 | 21.0-40.2 | 33.0-40.4 | 33.2-38.0 | 38.2-42.2 | - | - | 27.8-33.2 | 34.4-44.0 |
| **2.** *T.* sp. | 8.8-12.8 | - | 28.6-31.8 | 25.8-32.6 | 15.4-17.2 | 14.4 | 32.2-41.8 | 39.2 | 36.0 | 39.8 | - | - | 32.4 | 40.8 |
| **3.** *T. cancriformis* | 12.8-16.3 | 15.4-15.6 | - | 2.6-12.4 | 27.4-33.2 | 26.4-29.4 | 43.8-55.6 | 39.6-42.4 | 36.0-38.4 | 43.8-46.2 | - | - | 34.8-39.6 | 37.4-39.8 |
| **4.** *T. mauritanicus* | 13.4-19.6 | 15.7-18.1 | 7.9-10.6 | - | 22.0-31.4 | 22.6-28.8 | 38.2-53.8 | 44.4-53.0 | 38.0-45.4 | 43.0-51.8 | - | - | 36.6-44.2 | 41.8-50.2 |
| **5.** *T. longicaudatus* | 9.5-14.5 | 11.3-11.6 | 12.9-15.4 | 12.8-17.1 | - | 2.0 | 30.8-39.4 | 40.8-42.2 | 36.4-37.6 | 41.4-42.6 | - | - | 36.2-37.6 | 39.8-41.2 |
| **6.** *T. newberryi* | 9.79-14.07 | 11.3-12.4 | 14.4-15.3 | 13.9-16.9 | 0.0-3.7 | - | 32.8-40.0 | 38.2 | 34.8 | 39.6 | - | - | 33.8 | 38.4 |
| **7.** *T. granarius* | 12.5-19.7 | 12.1-19.2 | 20.1-21.8 | 17.0-21.7 | 14.6-17.8 | 15.5-17.4 | - | 41.8-48.2 | 42.0-54.0 | 45.8-52.6 | - | - | 33.6-41.8 | 47.0-52.8 |
| **8.** *L. viridis.* | 17.1-20.7 | 18.0-18.9 | 19.1-19.8 | 18.5-21.4 | 18.6-21.1 | 19.6-21.6 | 14.6-19.1 | - | 17.0 | 21.4 | - | - | 15.2 | 12.4 |
| **9.** *L. a. apus* | 14.9-22.6 | 16.9-17.4 | 16.6-17.3 | 18.6-22.4 | 16.9-19.6 | 18.0-19.1 | 18.7-20.7 | 12.8-13.4 | - | 16.0 | - | - | 10.4 | 10.4 |
| **10.** *L. a. lubbocki* | 16.1-20.7 | 18.6-19.1 | 17.0-17.7 | 17.2-20.0 | 16.4-17.9 | 16.9-18.4 | 18.8-19.7 | 15.3-16.3 | 15.8-16.9 | - | - | - | 20.6 | 22.2 |
| **11.** *L. couesii* | 19.7-23.6 | 22.4-22.7 | 16.9 | 19.4-21.4 | 18.6-20.6 | 19.6-20.6 | 20.6-20.8 | 10.5-11.0 | 15.6-16.0 | 15.4-15.8 | - | - | - | - |
| **12.** *L.* sp. | 19.4-25.5 | 21.5-22.6 | 20.0-20.6 | 20.6-23.8 | 19.3-21.8 | 19.9-22.3 | 20.0-22.2 | 11.4-12.5 | 16.1-17.4 | 17.3-18.7 | 7.7-8.1 | - | - | - |
| **13.** *L. lemmoni* | 16.8-19.9 | 16.1 | 18.0-18.2 | 17.3-20.1 | 17.4-18.8 | 18.1-18.6 | 17.6-19.2 | 11.9-12.2 | 15.3-15.6 | 16.1-16.6 | 15.6 | 16.5-17.0 | - | 15.4 |
| **14.** *L. arcticus* | 19.2-22.2 | 20.3-20.9 | 20.1-20.6 | 19.8-22.5 | 18.7-20.6 | 19.6-21.1 | 21.1-21.9 | 12.9-13.6 | 16.4-17.1 | 14.9-15.5 | 11.1-11.3 | 10.2-11.0 | 15.7-15.9 | - |
