# Supplementary material for: Toward a Global Phylogeny of the “Living Fossil" Crustacean Order of the Notostraca
Source: PLoS One. 2012 Apr 18;7(4):e34998. doi: 10.1371/journal.pone.0034998 (PMC3329532; doi:10.1371/journal.pone.0034998)
Supplement: Table S4 — Uncorrected p distance matrix (min.-max.) between investigated notostracan lineages based on COI (below diagonal) and 12S rRNA (above diagonal) genes. Empty cells indicate that sequence information was unavailable. (DOCX) [file pone.0034998.s005.docx]

**Table S4.** *Uncorrected p distance matrix (min.-max.) between investigated notostracan lineages based on COI (below diagonal) and 12S rRNA (above diagonal) genes. Empty cells indicate that sequence information was unavailable.*

|  | **1** | **2** | **3** | **4** | **5** | **6** | **7** | **8** | **9** | **10** | **11** | **12** | **13** | **14** |
| --- | --- | --- | --- | --- | --- | --- | --- | --- | --- | --- | --- | --- | --- | --- |
| **1.** *T. australiensis* | - | 6.3-8.9 | 9.9-13.7 | 9.4-13.3 | 6.3-8.6 | 5.4-8.9 | 8.7-14.1 | 12.5-14.4 | 12.5-13.8 | 13.8-14.8 | - | - | 10.9-12.5 | 12.9-14.8 |
| **2.** *T.* sp. | 10.6-14.6 | - | 11.1-12.0 | 10.2-12.1 | 6.6-7.2 | 6.6 | 12.0-14.6 | 14.2 | 13.3 | 14.2 | - | - | 12.3 | 14.6 |
| **3.** *T. cancriformis* | 14.6-17.6 | 16.9-17.1 | - | 1.3-5.4 | 10.8-12.4 | 10.4-11.4 | 15.2-17.8 | 14.2-14.9 | 13.3-14.0 | 15.2-15.9 | - | - | 13 | 13.7-14.3 |
| **4.** *T. mauritanicus* | 15.2-19.9 | 17.1-19.0 | 9.5-12.0 | - | 9.0-11.9 | 9.3-11.2 | 14.1-17.4 | 15.4-17.3 | 13.8-15.8 | 15.1-17.0 | - | - | 13.5-15.4 | 14.8-16.7 |
| **5.** *T. longicaudatus* | 11.3-15.9 | 13.1-13.4 | 14.6-16.8 | 14.5-18.0 | - | 0.9 | 11.8-14.0 | 14.6-14.9 | 13.4-13.7 | 14.6-15.0 | - | - | 13.3-13.6 | 14.3-14.6 |
| **6.** *T. newberryi* | 11.6-15.5 | 13.0-14.1 | 15.9-16.6 | 15.3-17.8 | 0-4.8 | - | 12.3-14.2 | 13.9 | 13,000 | 14.2 | - | - | 12.6 | 13.9 |
| **7.** *T. granarius* | 14.3-20.1 | 16.0-19.8 | 20.5-21.5 | 18.2-21.3 | 16.2-18.7 | 16.9-18.3 | - | 14.8-16.4 | 14.9-17.7 | 15.8-17.4 | - | - | 12.6-14.8 | 16.1-17.4 |
| **8.** *L. viridis* | 18.3-21.0 | 19.0-19.7 | 19.8-20.3 | 19.4-21.5 | 19.6-21.3 | 20.2-21.7 | 16.0-19.9 | - | 7.2 | 8.8 | - | - | 6.5 | 5.3 |
| **9.** *L. a. apus* | 16.4-22.4 | 18.2-18.5 | 18.0-18.5 | 19.4-22.2 | 18.2-20.3 | 19.0-19.9 | 19.6-21.0 | 14.5-15.0 | - | 6.9 | - | - | 4.7 | 4.7 |
| **10.** *L. a. lubbocki* | 17.5-20.8 | 19.4-19.7 | 18.2-18.7 | 18.3-20.5 | 17.6-18.7 | 18.0-19.0 | 19.8-20.1 | 16.5-17.4 | 16.9-17.8 | - | - | - | 8.4 | 9 |
| **11.** *L. couesii* | 20.3-23.1 | 22.2-22.4 | 18.2 | 20.1-21.5 | 19.6-21.0 | 20.3-21.0 | 20.8-21.2 | 12.0-12.5 | 16.9-17.3 | 16.8-17.1 | - | - | - | - |
| **12.** *L.* sp | 20.1-24.2 | 21.7-22.4 | 20.5-21.3 | 21.0-23.1 | 20.1-21.9 | 20.6-22.2 | 20.5-22.2 | 12.9-13.9 | 17.3-18.3 | 18.1-19.2 | 9.3-9.7 | - | - | - |
| **13.** *L. lemmoni* | 18.0-20.5 | 17.5 | 19.0-19.2 | 18.5-20.6 | 18.5-19.6 | 19.0-19.4 | 18.7-19.9 | 13.6-13.9 | 16.4-16.8 | 17.3-17.6 | 16.9 | 17.6-18.0 | - | 6.6 |
| **14.** *L. arcticus* | 19.9-22.0 | 20.6-21.0 | 20.6-21.0 | 20.5-22.4 | 19.6-21.0 | 20.2-21.3 | 21.0-21.9 | 14.1-14.8 | 17.5-18.0 | 16.0-16.5 | 12.5-12.7 | 11.8-12.5 | 16.9-17.1 | - |
