# Supplementary material for: Toward a Global Phylogeny of the “Living Fossil" Crustacean Order of the Notostraca
Source: PLoS One. 2012 Apr 18;7(4):e34998. doi: 10.1371/journal.pone.0034998 (PMC3329532; doi:10.1371/journal.pone.0034998)
Supplement: Text S1 — Overview of currently accepted Notostraca species and their known distributions. (DOC) [file pone.0034998.s006.doc]

**Text S1**: *Overview of currently accepted Notostracan species and their known distributions*

Currently accepted species in *Lepidurus* include the circumarctic *L. arcticus* (Pallas, 1793) [3,23] and the widespread *L. apus* (Linnaeus, 1758), including three subspecies: *L. apus lubbocki* from Italy and Northern Africa [81,82], *L. apus apus* from the rest of Europe [83] and *L. apus viridis*, restricted to Australia and New Zealand [18]. South American populations of this species are currently being redescribed (D.C. Rogers, EcoAnalysts, Inc., personal communication). North American species include *L. packardi* Simon, 1886*, L. cryptus* Rogers, 2001*, L. lemmoni* Holmes, 1894, *L. bilobatus* Packard, 1883) and *L. couesii* Packard, 1875 [16,84]. An ongoing morphological study supports that presumed *L. couesii* populations from the Mediterranean basin [84], Eastern Europe [85] and Mongolia [86] represent different species [16]. *L. mongolicus* has been described from Mongolia [87]. In Africa, *Lepidurus* is only known from fossilised remains from the Triassic of South Africa [36]. An extensive search of published literature revealed no records of current *Lepidurus* populations in Africa or South America.

Even though *T. cancriformis* (Bosc, 1801) is referred to as the European tadpole shrimp, its distribution extends beyond Europe, including northern Africa [13,88], Namibia [89], Iran, northern India and Japan [90,91]. Previously considered a subspecies of *T. cancriformis*, *T. mauritanicus* is now recognized as a proper species with populations on both sides of the Mediterranean in Spain, Portugal and Morocco [22,42]. On the other hand, *T. longicaudatus* (le Conte, 1846) is mainly restricted to the Americas, having been reported in large parts of the USA, Canada, Mexico, the Caribbean and the Galapagos Islands [21,35,51,63]. Pereira & Pereira [92] mention observations of *T. longicaudatus* on the South American continent, though these are poorly documented. Additionally, its presence has been confirmed in Japan [2,90]. A third species, *T. australiensis* (Spencer & Hall, 1895), is found in various temporary waters, such as pans, across the Australian continent [18,49]. Recently, *Triops* populations have been discovered from temporary rock pools on a number of Australian inselbergs (Balan Rock, Walga Rock, Baladonia Rock and Wonberna Rock) as well as a newly discovered population on Uluru (Ayers Rock) and a population from a saline lake (Lake Carey, Western Australia), all of which are included in this study. Remarkably, *Triops* populations from Madagascar have been described as a subspecies of *T. australiensis*: *T. australiensis sakalavus* [18,93]. *T. granarius* (Lucas, 1864) has a highly disjunct distribution, including Japan, China [90] and both northern and southern Africa [49]. Lastly, *T. newberryi* Thomas, 1921 is limited in its distribution to the west coast and the South-West of the United States [19,51,94].

**References**

81. Kuller Z, Gasith A (1996) Comparison of the hatching process of the tadpole shrimps *Triops cancriformis* and *Lepidurus apus lubbocki* (Notostraca) and its relation to their distribution in rain-pools in Israel. Hydrobiologia 335: 147-157.

82. Samraoui B, Chakri K, Samraoui F (2006) Large branchiopods (Branchiopoda: Anostraca, Notostraca and Spinicaudata) from the salt lakes of Algeria. Journal of limnology: 83-88.

83. Brtek J, Thiery A (1995) The geographic distribution of the European branchiopods(Anostraca, Notostraca, Spinicaudata, Laevicaudata). Hydrobiologia 298: 263-280.

84 Lynch JE (1972) *Lepidurus couesii* Packard (Notostraca) redescribed with a discussion of specific characters in the genus. Crustaceana 23: 43-49.

85. Thiéry A, editor (1996) Branchiopodes I. Ordres des Anostracés, Notostracés, Spinicaudata et Laevicaudata (Anostraca Sars,1867 − Notostraca Sars, 1867 − Spinicaudata Linder, 1945 − Laevicaudata Linder, 1945). Paris: Masson. 287-351p.

86. Brtek J, Forró L, Ponyi JE (1984) Contributions to the knowledge of the Branchiopoda (Crustacea) fauna of Mongolia. Annales Historico-Naturales Musei Nationalis Hungarici 76: 91-99.

87. Vekhov NV (1992) *Lepidurus mongolicus* sp. nov., a new species of tadpole shrimp (Crustacea, Notostraca, Triopsidae) from semideserts of Central Asia (Mongolia). Arthropoda Selecta 1: 89-93.

88. Thiery A (1991) Multispecies coexistence of branchiopods (Anostraca, Notostraca and Spinicaudata) in temporary ponds of Chaquia plain (Western Morocco) - sympatry or syntopy between usually allopatric species. Hydrobiologia 212: 117-136.

89. Barnard KH (1929) A revision of the South African Branchiopoda (Phyllopoda). Annals of the South African Museum 29: 191-272.

90. Takahashi F (1994) Use of the tadpole shrimp (*Triops* ssp.) as a biological control agent in the paddy fields of Japan. Hiroshima, Japan: Hiroshima University.

91. Golzari A, Khodabandeh S, Seyfabadi J (2009) Some biological characteristics of tadpole shrimp, *Triops cancriformis*, from seasonal pools of West Azarbaijan (Iran). Journal of Agricultural Science and Technology 11: 81-90.

92. Pereira G, Pereira M (1980) *Dendrocephalus geayi* en Venezuela: Redescripción y notas bioecológicas. Acta Científica Venezolana 31: 246.

93. Nobili G (1905) Descrizione di un nuevo *Apus* di Madagascar. Boll Mus Torino 20: 1-4.

94. Tianyun SU, Mulla MS (2002) Introduction and establishment of tadpole shrimp *Triops newberryi* (Notostraca: Triopsidae) in a date garden for biological control of mosquitoes in the Coachella Valley, southern California. Journal of Vector Ecology 27: 138-148.
