# Supplementary material for: Toward a Global Phylogeny of the “Living Fossil" Crustacean Order of the Notostraca
Source: PLoS One. 2012 Apr 18;7(4):e34998. doi: 10.1371/journal.pone.0034998 (PMC3329532; doi:10.1371/journal.pone.0034998)
Supplement: Text S2 — DNA isolation, molecular markers, polymerase chain reaction and sequencing. (DOC) [file pone.0034998.s007.doc]

**Text S2: *DNA isolation, molecular markers, polymerase chain reaction and sequencing***

Up to six tadpole shrimp specimens from each geographical location were dissected in order to obtain phyllopod tissue for DNA extraction. Genomic DNA was extracted from tissue using the NucleoSpin**®** extraction kit for individual samples (Macherey-Nagel). The mitochondrial COI DNA region was amplified by means of a polymerase chain reaction (PCR) using forward (5’ GGT CAA CAA ATC ATA AAG ATA TTG G 3’) and reverse (5’ TAA ACT TCA GGG TGA CCA AAA AAT CA 3’) invertebrate primers [95]. For amplification of the 12S region, the forward (5’ ATG CAC TTT CCA GTA CAT CTA C 3’) and reverse (5’ AAA TCG TGC CAG CCG TCG C 3’) were administered [66]. The reaction volume of 25 µL contained 2µl of template DNA, 2mM of MgCl2, 1mM 10 X reaction buffer, 0.2mM dNTPs, 0.4µM of each primer and 1,1U TAQ polymerase. The cycle settings were modified from [96] with an initial denaturation for 3min at 94°C, followed by five liberal amplification cycles (denaturation for 1min at 94°C, annealing for 1.5min at 45°C and elongation for 1.5min at 72°C) and 35 more rigid cycles (denaturation for 1min at 94°C, annealing for 1.5min at 50°C and elongation for 1.5min at 72°C), followed by a final elongation of 6min at 72°C. Reaction contaminants were removed from the samples using the NucleoFast**®** 96 PCR Clean-Up kit (Macherey-Nagel). Samples were sequenced with the Big Dye Terminator 3.1 kit (Applied Biosystems), following a 1/8 dilution of the Big Dye Terminator sequencing protocol, using the same primers [95,96]. Finally the products were run on an ABI PRISM 3130 Avant Genetic Analyzer automated sequencer (Applied Biosystems).

**References**

95. Folmer O, Black M, Hoeh W, Lutz R, Vrijenhoek R (1994) DNA primers for amplification of mitochondrial cytochrome c oxidase subunit I from diverse metazoan invertebrates. Molecular Marine Biology and Biotechnology 5: 294-299.

96. Adamowicz SJ, Hebert PDN, Marinone MC (2004) Species diversity and endemism in the *Daphnia* of Argentina: a genetic investigation. Zoological Journal of the Linnean Society 140: 171-205.
